# Supplementary material for: NPM1-Mutated Patient-Derived AML Cells Are More Vulnerable to Rac1 Inhibition
Source: Biomedicines. 2022 Aug 4;10(8):1881. doi: 10.3390/biomedicines10081881 (PMC9405324; doi:10.3390/biomedicines10081881)
Supplement: Supplementary file 1 [file biomedicines-10-01881-s001.zip › biomedicines-1815141-supplementary.pdf]

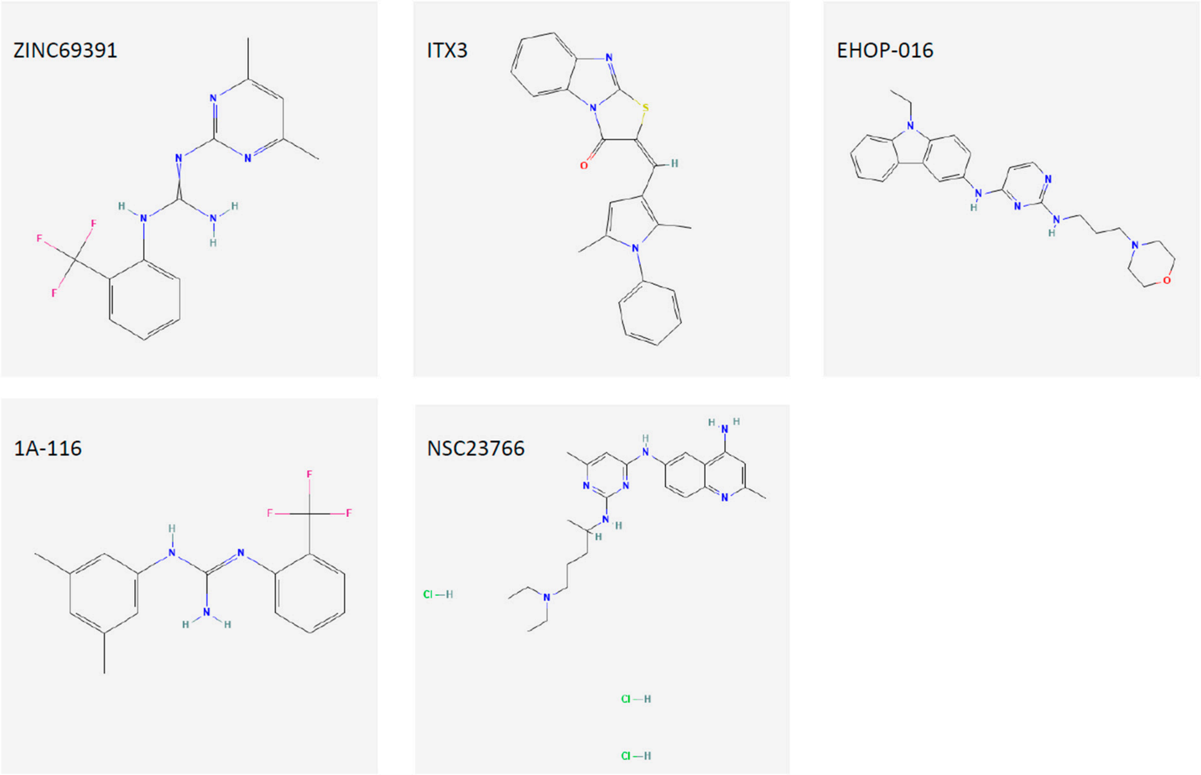


**Figure S1: Chemical structure of the Rac1 inhibitors.** 2D images of the chemical structures are downloaded from PubChem (pubchem.ncbi.nlm.nih.gov).

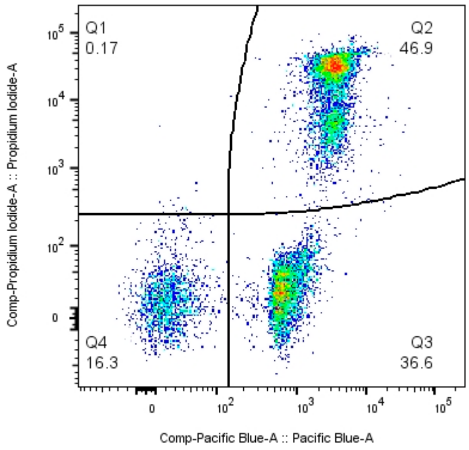


**Figure S2**: **Gating strategy.** Example of gating strategy of the Annexin V/Propidium iodine (PI) assay from one patient sample. The square "Q4" includes single-cell events considered negative for both Pacific Blue (Annexin V) on the x-axis and PI staining on the y-axis. "Q4" cells are regarded as viable cells. "Q3" includes Pacific blue positive and PI negative cells, considered apoptotic cells. Cells in "Q2" are considered necrotic cells, staining positive for both markers.

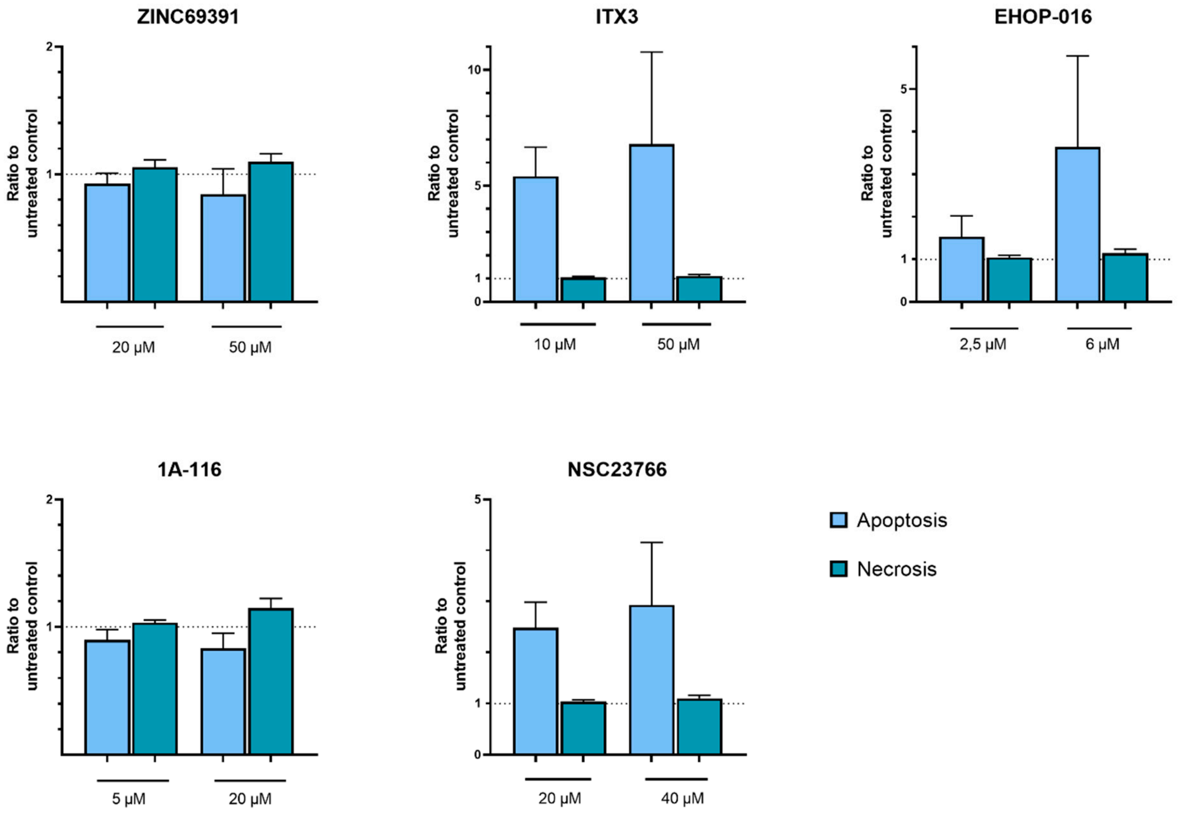


**Figure S3. Induction of apoptosis and necrosis separated for each Rac1 inhibitor.**

Flow cytometric assay to evaluate apoptosis and necrosis after 48 h treatment of 68 patient-derived AML samples with Rac1 inhibitors. There is one graph for each Rac1 inhibitor. The results are presented as a ratio to untreated control, set to 1 (dotted line). Columns are medians with 95% CI as an error. Blue columns indicate apoptosis; green columns indicate necrosis. The concentration of the inhibitors is given on the x-axis. The induction of apoptosis is the main contribution of ITX3, EHOP-016, and NSC23766. Necrosis is the main contribution of the compounds ZINC69391 and 1A-116.
